# Supplementary material for: The Evolution and Characterization of the RNA Interference Pathways in Lophotrochozoa
Source: Genome Biol Evol. 2024 May 7;16(5):evae098. doi: 10.1093/gbe/evae098 (PMC11114477; doi:10.1093/gbe/evae098)
Supplement: evae098_Supplementary_Data [file evae098_supplementary_data.zip › Supplementary_materials.zip!/selected_miRNAs/NoGe_selected_miRNAs/novel_miRNA_families_pdf_files/NMRB01000231.1_Notospermus_geniculatus_strain_Ushimado_scaffold231,_whole_genome_shotgun_sequence_7807.pdf]

Provisional ID	:	NMRB01000231.1_Notospermus_geniculatus_strain_Ushimado_scaffold231,_whole_genome_shotgun_sequence_7807
Score total	:	1243.9
Score for star read(s)	:	3.9
Score for read counts	:	1234.9
Score for mfe	:	2.1
Score for randfold	:	0
Score for cons. seed	:	3
Total read count	:	2434
Mature read count	:	2425
Loop read count	:	0
Star read count	:	9

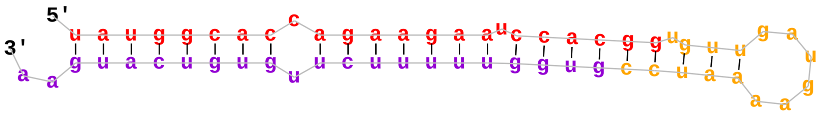

Genome coordinates: NMRB01000231.1 438872-438932

pre-miRNA sequence:
TATGGCACCAGAAGAATCCACGGTATGGCACCAGAAGAATCCACGGTGTTGATGAAAATCCGTGGTTTTTCTTGTGTCATGAA

Mature sequence: TATGGCACCAGAAGAATCCACGG

BLAST results for the pre-miRNA sequence against the *Lineus longissimus* genome assembly:

**Scaffold**	**Start**	**Stop**	**Identity**	**e-value**
OU342999.1 1	1850896	1850953	93.103	1.74e-16

```
Nge_novel_miRNA     TATGGCACCAGAAGAATCCACGGTGTTGATGAAAATCCGTGGTTTTTCTTGTGTCATG
Llo_genome          TATGGCACCAGAAGAATCCACGGTGTTGATGATAATCTGTGATTGTTCTTGTGTCATG
```
